# Factors associated with scientific production conditions among dental students from the Peruvian capital: an analysis under a multivariable regression model

**DOI:** 10.1186/s12909-024-06171-x

**Published:** 2024-10-15

**Authors:** Yanira Gutierrez-Quevedo, Marysela Ladera-Castañeda, Miriam Castro-Rojas, José Huamani-Echaccaya, Leysi Romero-Velásquez, Rosa Aroste-Andía, Luis Cervantes-Ganoza, César Cayo-Rojas

**Affiliations:** 1 Postgraduate School, Universidad Nacional Federico Villarreal, Lima, Peru; 2 School of Stomatology, Universidad Privada San Juan Bautista, Ica, Peru; 3 Faculty of Stomatology, Universidad Inca Garcilaso de la Vega, Lima, Peru

**Keywords:** Scientific production, Scientific research, Scientific publication, Dental education, Dentistry, Peru

## Abstract

**Background:**

The purpose of scientific production is to synthesize and capture research for eventual publication. In Peru, scientific production at the undergraduate level is relatively limited. The purpose of this study was to identify the factors associated with scientific production conditions among dental students from the Peruvian capital.

**Methods:**

This cross-sectional and analytical study evaluated 338 dental students from the Peruvian capital using a questionnaire composed of 15 questions on conditions for scientific production. Pearson’s chi-square test and Fisher’s exact test were used for bivariate analysis. To evaluate the influential variables, the adjusted Poisson regression model with robust variance using the adjusted prevalence ratio (APR) was employed. The significance level was *p* < 0.05.

**Results:**

A total of 17.8% of the students carried out research studies, while 1.5% published scientific articles. Conditions for scientific production were considered unfavorable in 28.4% of the cases, while 50.6% were classified as somewhat favorable and 21% as favorable. Students who dedicated < 2 h and ≥ 2 h per week to research were 3.04 and 3.84 times more likely to have favourable conditions for scientific production, respectively, compared to those who had no time for it (APR = 3.04, 95% CI: 1.02–9.03 and APR = 3.84, 95% CI: 1.13–13.02; respectively).

**Conclusion:**

A minority of dental students reported favorable conditions for scientific production. On the other hand, students with more weekly time for research are more likely to have favourable conditions for scientific production compared to those with no time.

**Supplementary Information:**

The online version contains supplementary material available at 10.1186/s12909-024-06171-x.

## Background

Scientific research is a fundamental means of generating knowledge about specific aspects of reality and contributing to society’s advancement through scientific and technological progress. Therefore, disseminating research results is crucial. In the training of health professionals such as dentists, research not only facilitates the development of critical thinking skills but it also enables the application of evidence-based dentistry [1].

The term “scientific production” refers to the process of synthesizing and expressing research in order to publish it. Two stages inextricably link to this process: research and publication. Nevertheless, at the undergraduate level, students frequently engage in research under the guidance or encouragement of their instructors. However, despite this opportunity, the majority of students do not ultimately publish their findings, and the quality of their work is often insufficient to achieve this goal [2].

While the development of a PhD is characterized by an advanced level of education and research, the purpose of which is to train researchers capable of creating new knowledge [3], it is equally crucial that research is an integral part of the training of undergraduate students, especially in the health sciences [4, 5]. Although undergraduate research is usually focused primarily on training students to read and interpret, rather than to conduct a research project. Therefore, it is essential that future professionals in this field develop critical thinking and oral and written communication skills from an early stage, in order to provide safe, efficient and competent clinical practice capable of addressing relevant health problems [4, 6]. It is evident that fostering interest in research and intellectual curiosity is of paramount importance for students, not only to facilitate learning, but also to encourage the application of critical thinking in real-life situations [6].

Some studies have indicated that undergraduate students can enhance their scientific production [7, 8]. However, the production of these students is relatively low in high-impact journals, with much of their research being published in non-indexed journals. This could be due to the low quality of research or lack of funding or other factors [9, 10]. It is therefore crucial that undergraduate students, in collaboration with experienced research professors, engage in the development of funded research projects as an integral component of their academic training. Such an approach would foster interest in the scientific method, allowing students to learn by doing while facing new challenges [11]. Such an experience would not only motivate them to generate high-quality scientific production, but would also prepare them to take on important challenges at postgraduate level, thus enabling them to reach their full potential [4, 5, 10].

Currently, scientific production is employed as a metric to assess the extent of research activity within any given professional field. According to the 2022 SCImago report, the countries with the highest overall scientific production were China, the United States, India, and the United Kingdom. The Latin American context identified Brazil, Mexico, Chile, and Colombia as the leading countries in terms of scientific production. Peru’s contribution to global scientific production ranked 62nd, securing a 6th place ranking in Latin America [12]. By 2022, the United States, India, and Brazil had emerged as the leading countries in scientific production in the field of dentistry worldwide. At the Latin American level, Peru (position 35), Brazil, and Chile were the countries with the highest scientific production in Scopus [12]. According to the Scimago Journal & Country Rank, from 1996 to 2022, the United States produced a total of 69,351 publications, Brazil produced 34,470 publications, and Peru produced 770 publications. The United States had 1,716,871 citations, Brazil had 583,480 citations, and Peru had 4,731 citations [12].

In Peru, Castro-Rodríguez et al. [13] and Pares-Ballasco et al. [14] reported student dental scientific production percentages of 3.5% and 2.4%, respectively. Additionally, dental students rated their knowledge of research and scientific production as deficient [13]. Researchers have conducted multiple studies to identify the elements that influence the scientific productivity of dentistry students. The findings indicate that academic factors are the primary contributors. The inclusion of writing and scientific production courses, together with the allocation of specific research time, has a favorable impact on scientific output [1]. Research suggests that the following factors increase the probability of scientific production in dental students: being in their final years of study, receiving peer support, participating in scientific organizations, and dedicating more than four hours a day to research [15].

Previous research in this field has been conducted in public universities, so this novel study examines the factors that influence the conditions of scientific production in a private Peruvian university (Universidad Privada San Juan Bautista) with just over 25 years of operation. In the Scimago Institutions Ranking 2024, this university is ranked 2nd in Peru (behind the Universidad Nacional Mayor de San Marcos, the Dean of America), 58th in Latin America, 80th in Latin America and 452nd in the world [16]. In light of the recent inclusion of this university in the Scimago Ranking from 2023, its possession of private investment resources, and its continued development of research infrastructure and computing resources, including high-quality computing equipment, high-speed networks, and increased access to specialised databases, as well as economic incentives and institutional recognition, which enable it to outperform Peruvian public universities that still have a limited budget to carry out these research activities [17–19], it is imperative to investigate the possible unfavorable conditions for scientific production and identify the limiting factors. In the event that these factors are indeed present, it would be prudent to design strategies to modify them and improve the conditions for the advancement of scientific production in undergraduate dentistry.

The purpose of this study was to identify the factors associated with scientific production conditions among dental students in the Peruvian capital. The null hypothesis was that there are no factors associated with these students’ scientific production conditions.

## Methods

### Study design

This analytical, observational, and cross-sectional study was conducted between April and May 2023 at the School of Stomatology of the Universidad Privada San Juan Bautista (UPSJB) in Lima, Peru, in accordance with the Strengthening the Reporting of Observational Studies in Epidemiology (STROBE) guidelines [20]. The UPSJB was founded on April 8, 1997 and the site of the School of Stomatology is located in the ‘Ex Hacienda Villa’, in the district of Chorrillos, south of Lima, Peru and its only campus is located 240 km south in Ica, Peru. This School of Stomatology appeared for the first time in the Scimago Institutional Ranking (SIR) in the year 2023, occupying the 5th place nationally considering public and private universities. By 2024 it ranked 1st as a private university and 2nd including public universities.

### Population and selection of participants

The study population consisted of a total of 395 dental students enrolled in the 2023–1 semester at the UPSJB, School of Stomatology. The study included the entire population, so no sample size calculation was required. The total target population was *N* = 338, with the following eligibility criteria taken into consideration:

Eligibility criteria (*N* = 356).

- Stomatology students enrolled from 1st to 5th year in 2023-1, at the Universidad Privada San Juan Bautista.
- Students of legal age (≥ 18 years).
- Students who attended classes during the recruitment period.
- Students who agreed to participate and gave informed consent to be part of the study.

Subsequently, the sample analysed was 338, due to 18 missing data.

### Variables

- Scientific production conditions were considered as the dependent variable. Its categories were unfavorable, somewhat favorable and favorable conditions. In the regression model, the category of interest was unfavorable conditions.
- The independent variable was time dedicated to research [1, 15] (the categories were: < 2 h per week / ≥ 2 h per week / None). In the regression model, the last category was taken as the reference for comparison in the analysis.
- The possible confounding variables were sex [1, 15] (the categories were: Male / Female); age group [21–23] (the categories were: ≤ 22 years / > 22 years, taking the median as the cut-off point); academic year [14, 15, 21] (the categories were: 1st year / 2nd year / 3rd year / 4th year / 5th year); participation in completed research work [18] (the categories were: Yes / No); publication: refers to any original article, review article and/or clinical report accepted for publication in any scientific journal [21] (the categories were: Yes / No); time devoted to clinical and academic activities [21] (the categories were: ≤ 15 h per week / > 15 h per week); work to generate economic income [1] (the categories were: Yes / No); and the number of courses taken in the UPSJB school of dentistry (the categories were: < 8 courses / ≥ 8 courses; taking the median as the cut-off point) and outside it (the categories were: One course / None) [1]. In the regression model, the last category was taken as the reference for comparison in the analysis.

### Instrument

An adapted questionnaire previously applied to Peruvian dental students was used [1]. The questionnaire consisted of 15 items related to the conditions for scientific production **[**Table 1**].**

**Table 1:** Initial questionnaire on the conditions for scientific production

Items
**Q1.** Do you subscribe to any national and/or international scientific journal?
**Q2.** Are you a member of any scientific society?
**Q3.** Are you a member of a research study group?
**Q4.** Are you a member of a multidisciplinary (multi-faculty) research group?
**Q5.** Have you participated in scientific poster competitions?
**Q6.** Have you attended national and/or foreign scientific congresses?
**Q7.** Have you organized national and/or foreign scientific events?
**Q8.** Did you have teaching assistance/advice for your scientific publications?
**Q9.** Did you have assistance/advice from your peers for your scientific publications?
**Q10.** Have you taken courses/instructions on scientific research?
**Q11.** Have you taken courses/instructions on scientific writing?
**Q12.** Have you taken courses/instructions on scientific publication/production?
**Q13.** Has the university and/or dental school motivated and encouraged you to publish scientific publications?
**Q14.** Have any professor motivated and encouraged you to publish scientific papers?
**Q15.** Does the university and/or dental school provide you with space and resources for your scientific publications?

Item Q2 was removed as it did not show response variability. Next, an item-total correlation matrix was applied, so item Q10 was eliminated, as its correlation value was less than < 0.35. An item-item correlation matrix was then constructed with a determinant of 0.020, which was acceptable. The factor analysis yielded acceptable values for the Kaiser-Meyer-Olkin test (0.819) and Bartlett’s sphericity (*p* < 0.001). Additionally, a principal components analysis with varimax rotation and Kayser normalization was performed, fixing the extraction in one factor, obtaining a factor loading of 60.57% of the variance explained in three variables. The communalities of all items oscillated between 0.362 and 0.814 (See supplementary material). Finally, Cronbach’s alpha was used to determine the reliability of the instrument, which yielded an acceptable internal consistency of 0.801 (95% CI: 0.768–0.831).

In order to identify the conditions for scientific production, the scores were classified into three categories: unfavorable (0 points), somewhat favorable (1 to 3 points), and favorable (4 points or more). The categories were established using the Stanones scale [mean ± 0.75 (standard deviation)] [24]. When the Livingston’s K^2^ coefficient was used to verify the two cut-off points for the three categories, it yielded acceptable values of 0.873 and 0.872, respectively [25].

### Procedure

Following the Ethics Committee’s approval (UNFV), a request was made to the School of Dentistry of the Universidad Privada San Juan Bautista for permission to apply the instrument in person. Once authorization had been granted, coordination was made with the classroom professors to provide the students with the questionnaire during the minutes prior to the start of classes, provided that they gave their voluntary, informed consent. The questionnaire and accompanying instructions were provided to the students who had consented to participate. A 10-minute period was allowed for the completion of the questionnaire. Additionally, participants were informed that they were at liberty to refrain from completing the questionnaire if they felt any discomfort in answering the questions. The institutional email address of the principal investigator was provided to the participants, as well as the contact information of the ethics committee in case they had any additional questions. The participants did not receive any form of compensation for their participation. The researchers then entered the data into a database for analysis. The information was exclusively accessible to the researchers, who safeguarded its confidentiality by storing it on a password-protected portable device. Any participant who requested them via email received the results upon the study’s conclusion.

### Data analysis

The data were analyzed using the Statistical Package for the Social Sciences (SPSS) version 28.0 (IBM, Armonk, New York, USA). For the descriptive analysis of qualitative variables, absolute and relative frequencies were employed. For the quantitative variable “age,” the mean was employed as a measure of central tendency, while the standard deviation served as a measure of dispersion. To ascertain the relationship between the independent variables and the questionnaire items, Pearson’s Chi-square test and Fisher’s exact test were employed for expected values below 5. In the multivariate analysis, a Poisson regression model with robust variance was employed, utilizing the adjusted prevalence ratio (APR) as the statistical measure. It was calculated by generalized linear modelling (GLM), using the logarithmic link function.

### Ethical aspects

The present study adhered to the ethical principles set forth in the Declaration of Helsinki, which include respect, freedom, nonmaleficence, and confidentiality [26]. The Ethics Committee of the Graduate School of the Universidad Nacional Federico Villarreal (UNFV) approved the study with resolution No. 008-CE-UIIE-EUPG-2022, dated August 23, 2022. Furthermore, the study requested the students to voluntarily provide their informed consent.

## Results

The complete response rate of the participants was 94.9%. The mean age of the participants was 22.8 ± 5.1 years. A total of 53.6% of the participants were aged ≤ 22 years. The total number of participants was 65.7% female. The academic years with the highest percentage of students were the second year (23.7%) and the third year (22.2%). A total of 17.8% of the students engaged in research studies. A total of 1.5% of the participants published an original article, review, or clinical report in a scientific journal, with one student publishing three articles (0.3%), another two articles (0.3%), and three students publishing one article each (0.9%). The five students indicated that the faculty had provided them with some form of recognition for their publications, with two students additionally reporting that a UPSJB Dental School member had also rewarded them. A total of 44.7% of respondents indicated that they do not allocate weekly time to research or scientific production. Nevertheless, 90.5% of the total reported spending at least some of their weekly time on clinical or academic activities. Additionally, 60.1% of students reported that they do not generate income. Finally, 55% of the total reported that they had taken eight or more courses within the UPSJB Dental School, while only 16.3% had taken one course outside it **[**Table 2**].**

**Table 2:** Characterization of the sociodemographic variables of dental students

Variable	Category	Frequency	Percentage
**V1.** Time dedicated to research	< 2 h per week	123	36.4
	≥ 2 h per week	64	18.9
	None	151	44.7
**V2.** Sex	Male	116	34.3
	Female	222	65.7
**V3.** Age group	≤ 22 years	181	53.6
	> 22 years	157	46.4
**V4**. Academic year	1st year	64	18.9
	2nd year	80	23.7
	3rd year	75	22.2
	4th year	67	19.8
	5th year	52	15.4
**V5.** Participation in completed research work	Yes	60	17.8
	No	278	82.2
**V6.** Publication (original article, review article and/or clinical report) in any scientific journal	Yes	5	1.5
	No	333	98.5
V6.1. How many research studies (original article, review article and/or clinical report) have you published?	None	333	98.5
	One	3	0.9
	Two	1	0.3
	Three	1	0.3
Q6.2. Has the university and/or dental school awarded you for your scientific publications?	Yes	5	1.5
	No	333	98.5
Q6.3. Have any professors awarded you for your scientific publications?	Yes	2	0.6
	No	336	99.4
**V7.** Time dedicated to clinical/academic activities	≤ 15 h per week	32	9.5
	> 15 h per week	306	90.5
**V8.** Works to generate income	Si	135	39.9
	No	203	60.1
**V9.** Number of courses taken at the UPSJB dental school	< 8 courses	152	45
	≥ 8 courses	186	55
**V10.** Number of courses taken outside the UPSJB dental school	One course	55	16.3
	None	283	83.7
	**Mean**	**Median**	**SD**
Age	22.9	22	5.1
Number of courses taken at the UPSJB dental school	7	8	2.5

*SD: Standard deviation.*

Conversely, it was observed that the conditions for scientific production among dental students were unfavorable in 28.4% (95% CI: 23.6 − 33.2%), somewhat favorable in 50.6% (95% CI: 45.3 − 55.9%), and favorable in 21.0% (95% CI: 16.7 − 25.4%) of the total number of participants.

A significant association was observed between being subscribed to a scientific journal and V1, V4, V5, and V9. Moreover, the majority of students who did not subscribe to a scientific journal did not spend any time on research either. The majority of students who did not belong to a research group in their field or a multidisciplinary group were also not interested in spending time on research. Participation in scientific poster competitions was found to be significantly associated with V1, V3, V4, V5, V6, V7 and V9; furthermore, those who never dedicated time to research were also not interested in participating in a scientific poster presentation. Attending scientific congresses was significantly associated with V1, V3, V4 and V5; furthermore, most students who attended these congresses dedicated 2 or more hours to research. The majority of students who never organized national and/or foreign scientific events were also not interested in spending time on research. The provision of teaching assistance/advice for scientific publications was found to be significantly associated with V1, V4, V5, and V6. Conversely, receiving advice from peers for the same purpose was significantly associated with V1 and V5. On the other hand, those students who did not receive advice from their professors and/or peers to prepare a scientific publication were also not interested in dedicating time to research. Taking courses on scientific writing was associated with V1, V3, V4, V5, V7 and V9, and taking courses on scientific publishing was significantly associated with V6 and the same variables, except V7. On the other hand, most of the students who did not take courses on scientific writing and publication were also not interested in dedicating time to research. In addition, V1, V3, V4, V5, and V6 were significantly associated with motivation or incentive received by university and/or dental school, and by any professor. However, those students who did not receive any kind of motivation or incentive from the university and/or dental school or the professors were also not interested in dedicating time to research. Finally, receiving space and resources for scientific publication from the university was significantly associated with V1, V3, V5 and V10. However, the most students who stated that the university did not provide them with resources and space for scientific production were also not interested in dedicating time to research **[**Table 3**].**

**Table 3:** Association of the conditions for scientific production with the sociodemographic variables of dental students

Variable	Category	Q1		Q3		Q4		Q5		Q6		Q7		Q8	
		Yes	No	Yes	No	Yes	No	Yes	No	Yes	No	Yes	No	Yes	No
		f (%)	f (%)	f (%)	f (%)	f (%)	f (%)	f (%)	f (%)	f (%)	f (%)	f (%)	f (%)	f (%)	f (%)
**V1**	< 2 h per week	8 (6.5)	115 (93.5)	1 (0.8)	122 (99.2)	1 (0.8)	122 (99.2)	2 (1.6)	121 (98.4)	67 (54.5)	56 (45.5)	2 (1.6)	121 (98.4)	3 (2.4)	120 (97.6)
	≥ 2 h per week	19 (29.7)	45 (70.3)	5 (7.8)	59 (92.2)	2 (3.1)	62 (96.9)	13 (20.3)	51 (79.7)	49 (76.6)	15 (23.4)	0 (0.0)	64 (100.0)	39 (60.9)	25 (39.1)
	None	5 (3.3)	146 (96.7)	0 (0.0)	151 (100.0)	0 (0.0)	151 (100.0)	0 (0.0)	151 (100.0)	55 (36.4)	96 (63.6)	0 (0.0)	151 (100.0)	0 (0.0)	151 (100.0)
	p*	< 0.001*		< 0.001*		0.045*		< 0.001*		< 0.001*		0.167		< 0.001*	
**V2**	Male	13 (11.2)	103 (88.8)	2 (1.7)	114 (98.3)	0 (0.0)	116 (100.0)	5 (4.3)	111 (95.7)	59 (50.9)	57 (49.1)	0 (0.0)	116 (100.0)	11 (9.5)	105 (90.5)
	Female	19 (8.6)	203 (91.4)	4 (1.8)	218 (98.2)	3 (1.4)	219 (98.6)	10 (4.5)	212 (95.5)	112 (50.5)	110 (49.5)	2 (0.9)	220 (99.1)	31 (14.0)	191 (86.0)
	p*	0.430		1.000		0.554		0.934		0.943		0.548		0.236	
**V3**	≤ 22 years	12 (6.6)	169 (93.4)	2 (1.1)	179 (98.9)	2 (1.1)	179 (98.9)	4 (2.2)	177 (97.8)	74 (40.9)	107 (59.1)	1 (0.6)	180 (99.4)	17 (9.4)	164 (90.6)
	> 22 years	20 (12.7)	137 (87.3)	4 (2.5)	153 (97.5)	1 (0.6)	156 (99.4)	11 (7.0)	146 (93.0)	97 (61.8)	60 (38.2)	1 (0.6)	156 (99.4)	25 (15.9)	132 (84.1)
	p*	0.056		0.422		1.000		0.033*		< 0.001*		1.000		0.069	
**V4**	1st year	2 (3.1)	62 (96.9)	0 (0.0)	64 (100.0)	0 (0.0)	64 (100.0)	0 (0.0)	64 (100.0)	15 (23.4)	49 (76.6)	0 (0.0)	64 (100.0)	2 (3.1)	62 (96.9)
	2nd year	3 (3.8)	77 (96.3)	3 (3.8)	77 (96.3)	1 (1.3)	79 (98.8)	1 (1.3)	79 (98.8)	27 (33.8)	53 (66.3)	0 (0.0)	80 (100.0)	7 (8.8)	73 (91.3)
	3rd year	6 (8.0)	69 (92.0)	1 (1.3)	74 (98.7)	0 (0.0)	75 (100.0)	1 (1.3)	74 (98.7)	45 (60.0)	30 (40.0)	0 (0.0)	75 (100.0)	10 (13.3)	65 (86.7)
	4th year	10 (14.9)	57 (85.1)	1 (1.5)	66 (98.5)	0 (0.0)	67 (100.0)	4 (6.0)	63 (94.0)	47 (70.1)	20 (29.9)	0 (0.0)	67 (100.0)	12 (17.9)	55 (82.1)
	5th year	11 (21.2)	41 (78.8)	1 (1.9)	51 (98.1)	2 (3.8)	50 (96.2)	9 (17.3)	43 (82.7)	37 (71.2)	15 (28.8)	2 (3.8)	50 (96.2)	11 (21.2)	41 (78.8)
	p*	0.002*		0.662		0.117		< 0.001**		< 0.001*		0.023*		0.020*	
**V5**	Yes	20 (33.3)	40 (66.7)	6 (10.0)	54 (90.0)	3 (5.0)	57 (95.0)	15 (25.0)	45 (75.0)	50 (83.3)	10 (16.7)	1 (1.7)	59 (98.3)	42 (70.0)	18 (30.0)
	No	12 (4.3)	266 (95.7)	0 (0.0)	278 (100.0)	0 (0.0)	278 (100.0)	0 (0.0)	278 (100.0)	121 (43.5)	157 (56.5)	1 (0.4)	277 (99.6)	0 (0.0)	278 (100.0)
	p*	< 0.001*		< 0.001*		0.005*		< 0.001**		< 0.001*		0.324		< 0.001*	
**V6**	Yes	1 (20.0)	4 (80.0)	0 (0.0)	5 (100.0)	1 (20.0)	4 (80.0)	2 (40.0)	3 (60.0)	5 (100.0)	0 (0.0)	0 (0.0)	5 (100.0)	5 (100.0)	0 (0.0)
	No	31 (9.3)	302 (90.7)	6 (1.8)	327 (98.2)	2 (0.6)	331 (99.4)	13 (3.9)	320 (96.1)	166 (49.8)	167 (50.2)	2 (0.6)	331 (99.4)	37 (11.1)	296 (88.9)
	p*	0.394		1.000		0.044*		0.017**		0.061*		1.000		< 0.001*	
**V7**	≤ 15 h per week	6 (18.8)	26 (81.3)	1 (3.1)	31 (96.9)	0 (0.0)	32 (100.0)	4 (12.5)	28 (87.5)	21 (65.6)	11 (34.4)	0 (0.0)	32 (100.0)	7 (21.9)	25 (78.1)
	> 15 h per week	26 (8.5)	280 (91.5)	5 (1.6)	301 (98.4)	3 (1.0)	303 (99.0)	11 (3.6)	295 (96.4)	150 (49.0)	156 (51.0)	2 (0.7)	304 (99.3)	35 (11.4)	271 (8.6)
	p*	0.102		0.452		1.000		0.043**		0.074		1.000		0.095	
**V8**	Yes	16 (11.9)	119 (88.1)	0 (0.0)	135 (100.0)	2 (1.0)	201 (99.0)	9 (6.7)	126 (93.3)	77 (57.0)	58 (43.0)	0 (0.0)	135 (100.0)	13 (9.6)	122 (90.4)
	No	16 (7.9)	187 (92.1)	6 (3.0)	197 (97.0)	1 (0.7)	134 (99.3)	6 (3.0)	197 (97.0)	94 (46.3)	109 (53.7)	2 (1.0)	201 (99.0)	29 (14.3)	174 (85.7)
	p*	0.222		0.085		1.000		0.105		0.053		0.519		0.204	
**V9**	< 8 courses	20 (13.2)	132 (86.8)	2 (1.3)	150 (98.7)	2 (1.3)	150 (98.7)	13 (8.6)	139 (91.4)	76 (50.0)	76 (50.0)	2 (1.3)	150 (98.7)	19 (12.5)	133 (87.5)
	≥ 8 courses	12 (6.5)	174 (93.5)	4 (2.2)	182 (97.8)	1 (0.5)	185 (99.5)	2 (1.1)	184 (98.9)	95 (51.1)	91 (48.9)	0 (0.0)	186 (100.0)	23 (12.4)	163 (87.6)
	p*	0.036*		0.649		0.590		< 0.001**		0.844		0.201		0.970	
**V10**	One course	30 (10.6)	253 (89.4)	1 (1.8)	54 (98.2)	1 (1.8)	54 (98.2)	12 (4.2)	271 (95.8)	148 (52.3)	135 (47.7)	1 (1.8)	54 (98.2)	39 (13.8)	244 (86.2)
	None	2 (3.6)	53 (96.4)	5 (1.8)	278 (98.2)	2 (0.7)	281 (99.3)	3 (5.5)	52 (94.5)	23 (41.8)	32 (58.2)	1 (0.4)	282 (99.6)	3 (5.5)	52 (94.5)
	p*	0.106		1.000		0.414		0.719		0.155		0.299		0.087	

Variable	Category	Q9		Q11		Q12		Q13		Q14		Q15			
		Yes	No	Yes	No	Yes	No	Yes	No	Yes	No	Yes	No		
		f (%)	f (%)	f (%)	f (%)	f (%)	f (%)	f (%)	f (%)	f (%)	f (%)	f (%)	f (%)		
**V1**	< 2 h per week	0 (0.0)	123 (100.0)	56 (45.5)	67 (54.5)	5 (4.1)	118 (95.9)	3 (2.4)	120 (97.6)	34 (27.6)	89 (72.4)	37 (30.1)	86 (69.9)		
	≥ 2 h per week	16 (25.0)	48 (75.0)	42 (65.6)	22 (34.4)	26 (40.6)	38 (59.4)	44 (68.8)	20 (31.3)	47 (73.4)	17 (26.6)	48 (75.0)	16 (25.0)		
	None	0 (0.0)	151 (100.0)	28 (18.5)	123 (81.5)	1 (0.7)	150 (99.3)	0 (0.0)	151 (100.0)	20 (13.2)	131 (86.6)	38 (25.2)	113 (74.8)		
	p*	< 0.001**		< 0.001*		< 0.001*		< 0.001*		< 0.001*		< 0.001*			
**V2**	Male	4 (3.4)	112 (96.6)	44 (37.9)	72 (62.1)	9 (7.8)	107 (92.2)	12 (10.3)	104 (89.7)	32 (27.6)	84 (72.4)	42 (36.2)	74 (63.8)		
	Female	12 (5.4)	210 (94.6)	82 (36.9)	140 (63.1)	23 (10.4)	199 (89.6)	35 (15.8)	187 (84.2)	69 (31.1)	153 (68.9)	81 (36.5)	141 (63.5)		
	p*	0.421		0.858		0.438		0.171		0.505		0.960			
**V3**	≤ 22 years	7 (3.9)	174 (96.1)	46 (25.4)	135 (74.6)	8 (4.4)	173 (95.6)	18 (9.9)	163 (90.1)	38 (21.0)	143 (79.0)	55 (30.4)	126 (69.6)		
	> 22 years	9 (5.7)	148 (94.3)	80 (51.0)	77 (49.0)	24 (15.3)	133 (84.7)	29 (18.5)	128 (81.5)	63 (40.1)	94 (59.9)	68 (43.3)	89 (56.7)		
	p*	0.421		< 0.001*		< 0.001*		0.024*		< 0.001*		0.014*			
**V4**	1st year	1 (1.6)	63 (98.4)	1 (1.6)	63 (98.4)	0 (0.0)	64 (100.0)	2 (3.1)	62 (96.9)	2 (3.1)	62 (96.9)	2 (3.1)	62 (96.9)		
	2nd year	5 (6.3)	75 (93.8)	5 (6.3)	75 (93.8)	3 (3.8)	77 (96.3)	8 (10.0)	72 (90.0)	8 (10.0)	72 (90.0)	27 (33.8)	53 (66.3)		
	3rd year	1 (1.3)	74 (98.7)	10 (13.3)	65 (86.7)	4 (5.3)	71 (94.7)	13 (17.3)	62 (82.7)	27 (36.0)	48 (64.0)	27 (36.0)	48 (64.0)		
	4th year	5 (7.5)	62 (92.5)	59 (88.1)	8 (11.9)	8 (11.9)	59 (88.1)	11 (16.4)	56 (83.6)	26 (38.8)	41 (61.2)	34 (50.7)	33 (49.3)		
	5th year	4 (7.7)	48 (92.3)	51 (98.1)	1 (1.9)	17 (32.7)	35 (67.3)	13 (25.0)	39 (75.0)	38 (73.1)	14 (26.9)	33 (63.5)	19 (36.5)		
	p*	0.175		< 0.001*		< 0.001*		0.008*		< 0.001		< 0.001			
**V5**	Yes	16 (26.7)	44 (73.3)	44 (73.3)	16 (26.7)	29 (48.3)	31 (51.7)	47 (78.3)	13 (21.7)	50 (83.3)	10 (16.7)	51 (85.0)	9 (15.0)		
	No	0 (0.0)	278 (100.0)	82 (29.5)	196 (70.5)	3 (1.1)	275 (98.9)	0 (0.0)	278 (100.0)	51 (18.3)	227 (81.7)	72 (25.9)	206 (74.1)		
	p*	< 0.001**		< 0.001*		< 0.001*		< 0.001*		< 0.001*		< 0.001*			
**V6**	Yes	1 (20.0)	4 (80.0)	4 (80.0)	1 (20.0)	3 (60.0)	2 (40.0)	4 (80.0)	1 (20.0)	5 (100.0)	0 (0.0)	4 (80.0)	1 (20.0)		
	No	15 (4.5)	318 (95.5)	122 (36.6)	211 (63.4)	29 (8.7)	304 (91.3)	43 (12.9)	290 (87.1)	96 (28.8)	237 (71.2)	119 (35.7)	214 (64.3)		
	p*	0.216		0.066		0.007*		0.001**		0.002**		0.061			
**V7**	≤ 15 h per week	3 (9.4)	29 (90.6)	21 (65.6)	11 (34.4)	5 (15.6)	27 (84.4)	6 (18.8)	26 (81.3)	12 (37.5)	20 (62.5)	16 (50.0)	16 (50.0)		
	> 15 h per week	13 (4.2)	293 (95.8)	105 (34.3)	201 (65.7)	27 (8.8)	279 (91.2)	41 (13.4)	265 (86.6)	89 (29.1)	217 (70.9)	107 (35.0)	199 (65.0)		
	p*	0.185		< 0.001*		0.207		0.420		0.322		0.093			
**V8**	Yes	5 (3.7)	130 (96.3)	49 (36.3)	86 (63.7)	12 (8.9)	123 (91.1)	13 (9.6)	122 (90.4)	34 (25.2)	101 (74.8)	50 (37.0)	85 (63.0)		
	No	11 (5.4)	192 (94.6)	77 (37.9)	126 (62.1)	20 (9.9)	183 (90.1)	34 (16.7)	169 (83.3)	67 (33.0)	136 (67.0)	73 (36.0)	130 (64.0)		
	p*	0.467		0.761		0.767		0.064		0.124		0.840			
**V9**	< 8 courses	7 (4.6)	145 (95.4)	75 (49.3)	77 (50.7)	22 (14.5)	130 (85.5)	20 (13.2)	132 (86.8)	52 (34.2)	100 (65.8)	50 (32.9)	102 (67.1)		
	≥ 8 courses	9 (4.8)	177 (95.2)	51 (27.4)	135 (72.6)	10 (5.4)	176 (94.6)	27 (14.5)	159 (85.5)	49 (26.3)	137 (73.7)	73 (39.2)	113 (60.8)		
	p*	0.920		< 0.001*		0.004*		0.720		0.116		0.227			
**V10**	One course	16 (5.7)	267 (94.3)	110 (38.9)	173 (61.1)	27 (9.5)	256 (90.5)	41 (14.5)	242 (85.5)	87 (30.7)	196 (69.3)	112 (39.6)	171 (60.4)		
	None	0 (0.0)	55 (100.0)	16 (29.1)	39 (70.9)	5 (9.1)	50 (90.9)	6 (10.9)	49 (89.1)	14 (25.5)	41 (74.5)	11 (20.0)	44 (80.0)		
	p*	0.085		0.170		0.917		0.483		0.433		0.006*			

f: frequency. * Based on Pearson’s chi-square (p < 0.05, significant association). **Based on Fisher’s exact test for expected values less than 5 (p < 0.05,* significant association). V1. Sex; V2. Age group; V3. Academic year; V4. Participation in completed research work; V5. Publication; V6. Time dedicated to research; V7. Time dedicated to clinical/academic activities; V8. Works to generate income; V9. Number of courses taken at the UPSJB dental school; V10. Number of courses taken outside the UPSJB dental school. Q1*,* Q3*,* Q4*,* Q5*,* Q6*,* Q7*,* Q8*,* Q9*,* Q11*,* Q12*,* Q13*,* Q14 and Q15: Final items of the questionnaire.*

The adjusted Poisson regression model with robust variance showed that students who dedicated < 2 h and ≥ 2 h per week to research were 3.04 and 3.84 times more likely to have favourable conditions for scientific production, respectively, compared to those who had no time for it (APR = 3.04, 95% CI: 1.02–9.03 and APR = 3.84, 95% CI: 1.13–13.02; respectively) **[**Table 4**].**

**Table 4:** Factors associated with favorable conditions for scientific production of dental students

Variable	Category	Crude model				Adjusted model		
		PR	95% CI			APR	95% CI	
			LL	UL			LL	UL
**V1.** Time dedicated to research	< 2 h per week	5.22	1.80	15.10		3.04*	1.02	9.03
	≥ 2 h per week	29.49	11.12	78.23		3.84*	1.13	13.02
	None	*Ref.*				*Ref.*		
**V2.** Sex	Male	1.09	0.70	1.70				
	Female	*Ref.*						
**V3.** Age group	≤ 22 years	0.53	0.35	0.82				
	> 22 years	*Ref.*						
**V4**. Academic year	1st year	0.06	0.01	0.23		0.15	0.04	0.51 [N.I]
	2nd year	0.19	0.09	0.38				
	3rd year	0.32	0.19	0.56				
	4th year	0.55	0.36	0.87				
	5th year	*Ref.*						
**V5.** Participation in completed research work	Yes	14.72	9.22	23.51		6.76	3.34	13.67 [N.I]
	No	*Ref.*						
**V6.** Publication (original article, review article and/or clinical report) in any scientific journal.	Yes	5.05	4.06	6.26				
	No	*Ref.*						
**V7.** Time dedicated to clinical/academic activities.	≤ 15 h per week	1.57	0.90	2.75				
	> 15 h per week	*Ref.*						
**V8.** Works to generate income	Yes	0.77	0.49	1.19				
	No	*Ref.*						
**V9.** Number of courses taken at the UPSJB dental school.	< 8 courses	1.58	1.04	2.40				
	≥ 8 courses	*Ref.*						
**V10.** Number of courses taken outside the UPSJB dental school.	One course	0.75	0.40	1.41				
	None	*Ref.*						

*Crude model: Prevalence ratio (PR) was obtained by Poisson simple regression with a robust estimator for each variable*, finding significant association (p < 0.05). Adjusted prevalence ratio (PAR) was performed by Poisson multiple regression with robust estimator of those variables that in the crude model had a significant association (p < 0.05). *The association between unfavorable conditions for scientific production and time dedicated to research,* adjusted for age group*,* academic year*,* participation in completed research work*,* publication and number of courses taken at the UPSJB dental school. [N.I]: not interpreted.*

## Discussion

Scientific research is of the utmost importance for the training of future dentists. Since 2014, Peruvian University Law 30,220 has prioritized training professionals with research competencies. For this reason, it is important to highlight the role of the vice-rectorate for research as the entity responsible for promoting university research through incentives, funding, coordination, dissemination, and promotion [27]. Despite the requirement for a research project (thesis) for graduation, most university students never publish their thesis, resulting in little or no scientific output. Tintaya [28] and Castro-Rodriguez et al. [29] in Peru reported frequencies of 3% (from 2013 to 2020) and 10% (from 2010 to 2018) in thesis published in scientific journals, carried out by dental students of the Universidad Privada San Juan Bautista and graduates of the Universidad Nacional Mayor de San Marcos as a graduation requirement.

The present study identified that of the 338 students surveyed, 17.8% had conducted research studies, yet only 1.5% had produced scientific publications. The results are comparable to those reported by Castro-Rodriguez et al. [13] and Pares-Ballasco et al. [14], who found percentages of student dental scientific production to be 3.5% and 2.4%, respectively. In Ecuador, Vallejo-López et al. [15] found that 10% of undergraduate dental students had published their research. This discrepancy is likely attributable to the disparity in research funding. In 2020, Ecuador allocated 0.4030% of its gross domestic product (GDP) for research activities, while Peru allocated 0.0985% [30]. In 2023, the Ecuadorian budget was higher, with a total of US$806 million, in contrast to the Peruvian budget of US$406 million [31].

The bivariate analysis of each variable with each item of the questionnaire revealed a significant association between publishing in scientific journals and being part of a multidisciplinary research group, participating in scientific poster competitions, receiving teaching assistance for their publications, taking courses on scientific publication or production, receiving motivation or incentive from the university or dental school, and receiving motivation or incentive from a professor. This finding is consistent with the results reported by Castro-Rodríguez [1], who identified a significant association between scientific publication and the conditions for scientific production. Participation in multidisciplinary groups with professors engaged in research and receiving their counsel would be associated with publishing, as it is an enriching experience for the student to have direct contact with research, production, and publication by experienced researchers. Participation in scientific poster competitions fosters familiarity with scientific research and dissemination, which in turn leads to publication. Students who take scientific publication or production courses are more likely to publish because they learn about the publication process. Receiving motivation or incentives from the university, dental school, or professor is associated with scientific publication, as novices in this field often perceive their studies as flawed or irrelevant. Receiving an incentive from a competent authority would provide the necessary strength or motivation to overcome these insecurities [32].

The present study did not identify a statistically significant association between scientific production and subscription to a national and/or international scientific journal. This may be due to the fact that not having this type of subscription does not necessarily represent a barrier to accessing quality information, since there are scientific publications that are freely accessible. Furthermore, as reported by Valladares-Garrido et al. [33], students use alternative resources, such as Sci-Hub or illicitly accessed academic accounts, to gain access to high-quality research.

In contrast to the findings of Mejía et al. [34] and Sánchez-Duque et al. [35], no significant association could be established between being a member of a scientific society and greater scientific production in the present study, as no students were reported to belong to any society. This study also demonstrates a lack of association between receiving assistance or advice from peers in the publication process and scientific production. The majority of students lacked experience in publishing scientific articles, which limited their ability to provide guidance and assistance to their peers. Similarly, there was no association between scientific production and participation in scientific research courses or instruction. These courses often prioritize theoretical aspects of research, which may not fully equip students with the procedural and attitudinal competencies necessary to publish scientifically. Offering students a personalized, step-by-step guide to the scientific method through practical scientific writing workshops, with a maximum of five students per researcher-professor, could reverse this scenario. Previous studies have shown that receiving guidance and enrolling in scientific writing and scientific production courses contribute to increased scientific output [1, 36]. Furthermore, the organization of scientific events and the attendance at scientific congresses did not show any association with the scientific production, possibly because these activities primarily focus on disseminating the latest research findings rather than delving into the complexities of scientific research and writing.

The present study demonstrated that 79% of dental students exhibited somewhat favorable to unfavorable conditions for scientific production. Additionally, multivariable statistical analysis revealed that devoting weekly time to research increased the likelihood of having favorable conditions for scientific production. Dedicating time to research on a regular basis could create habits that help students become familiar with the process of scientific research and publication. It would also allow them to develop competencies and improve skills such as information search, data analysis, or scientific writing. Similarly, Vallejo-López et al. [15] identified a lower scientific production in Ecuadorian students from the first year of dentistry who dedicate less than 2 h to research. Castro-Rodrguez [1] and Pares-Ballasco et al. [14] also found a significant association between dental students’ scientific production and the time dedicated to study.

Carhuancho-Mendoza et al. [37] found that working to generate income is associated with a lower probability of doing research and scientific production. This is because students who work prioritize their time to generate income and study, leaving the task of researching in the background. This is inconsistent with the present research, as this factor was not significantly associated with scientific production. This may be because, unlike Carhuancho-Mendoza et al. [37], this study used a multivariable regression analysis with robust variance. This demonstrates that linking variables in a bivariable analysis does not necessarily imply that they cause or influence each other. Regarding the number of courses taken at the dental school, Corrales-Reyes et al. [38] identified it as a limiting factor in scientific production. This is because students who enroll in more courses tend to experience an academic overload, with courses primarily focusing on acquiring knowledge about their profession and clinical practice to develop procedural skills, often neglecting the development of research skills, which are equally important for professional training. The present study did not find such an association, possibly because the academic plan of most Peruvian universities focuses not only on knowledge of the professional career but also on the development of scientific competencies, with courses related to research throughout the career. Some universities in Peru’s capital, such as the Universidad Privada San Juan Bautista, offer courses in systematization and statistical methods, research methodology, and the writing and publication of scientific articles. Another example is the Universidad Peruana de Ciencias Aplicadas, which offers courses in management, scientific information search, and scientific research methodology. A final example is the Universidad Científica del Sur, where general statistics and methodology for research in dentistry are taught. At all three universities, the degree program culminates in the completion of a thesis project, which can also take the form of a scientific article [39–41].

This study is significant because it provides a current overview of scientific production in dentistry at a university that did not appear in the Scimago ranking in 2022. However, by 2023, it had risen to the top 5 [42] and is currently in the top 2 in Peruvian scientific production according to the Scimago Institutional Ranking 2024 [16]. Despite the university’s reported low percentages of scientific production and unfavorable conditions, the rate of scientific production in the dental school is increasing. This may be the result of successive curriculum changes that aim to integrate subjects with competencies in dental research and social responsibility. This is reflected in recent updates to the academic plan. In the 2016 academic plan, the research courses were Biostatistics, Research Methodology, and Thesis Plans I and II [43]. The 2021 academic plan introduced a more comprehensive and research-oriented proposal, covering topics such as statistical methods, scientific search tools, research methodology, scientific article interpretation, writing scientific articles, and thesis I and II [44]. The current curricular plan prioritizes the publication of research results, offering courses like Systematization and Statistical Methods, Research Methodology, Writing and Publication of Scientific Articles I and II, Thesis Workshop, and Research Work [39, 45]. Research subjects, particularly the thesis and scientific publication courses taught by experienced researchers recognized by the National Council of Science, Technology, and Technological Innovation (CONCYTEC) [46], suggest an improvement in scientific production quality. Furthermore, the dental school boasts a research team with extensive experience in methodology, statistics, and high-impact scientific publications. This team is responsible for evaluating thesis proposals to ensure that they are relevant and fully justified. On the other hand, we acknowledge that University Law 30,220’s proposed reforms, which, unlike in previous years, emphasize the need for universities to meet certain fundamental criteria for quality education through licensing, may have spurred this surge in scientific output [47, 48]. This process now requires the mastery of another language, preferably English, at least at an intermediate level, the completion of a research project to obtain the degree and the presentation of a thesis to obtain the degree. Furthermore, Law 30,220 mandates that universities prioritize not only research but also scientific publication. It also stipulates that universities must employ research professors recognized by CONCYTEC, who represent at least 5% of the total teaching staff. Additionally, all university professors are required to possess at least a master’s degree, which facilitates their alignment with research guidelines [47].

This study was subject to certain limitations, including the inability to access the entire student body due to absences from classes and incomplete responses to questionnaires. Similarly, generalizing the results to all of Peru is not possible due to the evaluation of students from a single university and a single city. However, the study establishes a basis for scrutinizing the adverse circumstances hindering high-quality scientific output in institutions ranked highly in national or international rankings. Another limitation was that the number of students who had participated in completed and published research was very low, so it was not possible to analyze the factors associated with these variables, but it did allow us to identify this recurrent problem in dental students, as occurs in other benchmark universities in Peru. Finally, the present study’s cross-sectional design precludes any assessment of the conditions and factors influencing scientific production over time. Therefore, we cannot determine whether the 2020 academic plan has brought about any changes compared to previous years.

It would be advisable for all emerging universities to develop their own scientific journals, thus providing a platform for students to gain experience in publishing. In addition, it is essential to encourage greater participation of students from the beginning of dental training in multidisciplinary research groups with the mentorship of experienced research professors, under institutional management that provides adequate resources and spaces to develop quality research, allowing them to publish their work in high impact indexed journals. On the other hand, it would be prudent to conduct a more comprehensive investigation that encompasses dental students from both public and private universities in Peru and other Latin American countries. This would entail an examination of the discrepancies between the academic curricula and the research emphasis across universities, with a particular focus on those that have demonstrated excellence in scientific output at the international level. Finally, as a recommendation, although the items in Table 1 of the supplementary material showed some acceptable psychometric indicators, they still require further study, as the correlation matrix showed low correlations between items and, in addition, the exploratory factor analysis showed four eigenvalues > 1, indicating the presence of more than one sub-score.

## Conclusion

Although one Peruvian dental school ranked in the top 1 as a private university in the Scimago 2024 Institutional Ranking, or in the top 2 when public universities were also considered, only a minority of dental students reported favorable conditions for scientific production. On the other hand, students with more weekly time for research are more likely to have favorable conditions for scientific production compared to those with no time.

## Electronic supplementary material

Below is the link to the electronic supplementary material.

Supplementary Material 1

## Data Availability

All data analyzed during this study are available from the corresponding author on reasonable request (cesarcayorojas@gmail.com).

## Abbreviations

CONCYTEC: National Council of Science, Technology, and Technological Innovation
CI: Confidence interval
APR: Adjusted Prevalence Ratio
STROBE: Strengthening the Reporting of OBservational studies in Epidemiology
SPSS: Statistical Package for the Social Sciences
UPSJB: Universidad Privada San Juan Bautista
