# Supplementary material for: Factors associated with scientific production conditions among dental students from the Peruvian capital: an analysis under a multivariable regression model
Source: BMC Med Educ. 2024 Oct 15;24:1148. doi: 10.1186/s12909-024-06171-x (PMC11481528; doi:10.1186/s12909-024-06171-x)
Supplement: Supplementary file 1 — Supplementary Material 1 [file 12909_2024_6171_MOESM1_ESM.docx]

**Supplementary material**

Item responses were dichotomous (Yes / No). In addition, question Q2 was eliminated for showing no variability, while Q10 was subsequently eliminated for showing a correlation of less than 0.350 with the total item **[Table 1].**

**Table 1. Total - Item correlation and communalities**

| **Questionnaire** | **Correlation*** | **Communalities** |
| --- | --- | --- |
| **Q1** | 0.926 | 0.381 |
| **Q3** | 0.985 | 0.629 |
| **Q4** | 0.992 | 0.654 |
| **Q5** | 0.964 | 0.553 |
| **Q6** | 0.572 | 0.389 |
| **Q7** | 0.995 | 0.772 |
| **Q8** | 0.903 | 0.811 |
| **Q9** | 0.962 | 0.746 |
| **Q10** | 0.220 |  |
| **Q11** | 0.704 | 0.672 |
| **Q12** | 0.926 | 0.626 |
| **Q13** | 0.892 | 0.685 |
| **Q14** | 0.767 | 0.556 |
| **Q15** | 0.712 | 0.442 |

* Based on tetrachoric correlation.

**Table 2. Item - item correlation matrix**

| **Questionnaire** | **Q1** | **Q3** | **Q4** | **Q5** | **Q6** | **Q7** | **Q8** | **Q9** | **Q11** | **Q12** | **Q13** | **Q14** | **Q15** |
| --- | --- | --- | --- | --- | --- | --- | --- | --- | --- | --- | --- | --- | --- |
| **Q1** | 1.000 | 0.600 | 0.760 | 0.309 | 0.724 | 0.820 | 0.119 | 0.213 | 0.599 | 0.000 | 0.169 | 0.506 | 0.589 |
| **Q3** | 0.600 | 1.000 | 0.270 | 0.360 | 0.930 | 0.410 | 0.680 | 0.380 | 0.890 | 0.600 | 0.710 | 0,860 | 0.890 |
| **Q4** | 0.760 | 0.270 | 1.000 | 0.570 | 0.960 | 0.160 | 0.810 | 0.590 | 0.940 | 0.760 | 0.830 | 0.920 | 0.940 |
| **Q5** | 0.309 | 0.360 | 0.570 | 1.000 | 0.852 | 0.680 | 0.415 | 0.027 | 0.774 | 0.309 | 0.457 | 0.711 | 0.767 |
| **Q6** | 0.720 | 0.930 | 0.960 | 0.850 | 1.000 | 0.980 | 0.660 | 0.840 | 0.210 | 0.720 | 0.630 | 0.330 | 0.230 |
| **Q7** | 0.820 | 0.410 | 0.160 | 0.680 | 0.980 | 1.000 | 0.860 | 0.690 | 0.960 | 0.820 | 0.870 | 0.950 | 0.960 |
| **Q8** | 0.120 | 0.680 | 0.810 | 0.410 | 0.660 | 0.860 | 1.000 | 0.390 | 0.510 | 0.120 | 0.050 | 0.410 | 0.500 |
| **Q9** | 0.280 | 0.380 | 0.590 | 0.030 | 0.840 | 0.690 | 0.390 | 1.000 | 0.760 | 0.280 | 0.430 | 0.700 | 0.760 |
| **Q11** | 0.600 | 0.890 | 0.940 | 0.770 | 0.210 | 0.960 | 0.510 | 0.760 | 1.000 | 0.600 | 0.470 | 0.130 | 0.010 |
| **Q12** | 0.000 | 0.600 | 0.760 | 0.310 | 0.720 | 0.820 | 0.120 | 0.280 | 0.600 | 1.000 | 0.170 | 0.510 | 0.590 |
| **Q13** | 0.170 | 0.710 | 0.830 | 0.460 | 0.630 | 0.870 | 0.050 | 0.430 | 0.470 | 0.170 | 1.000 | 0.370 | 0.460 |
| **Q14** | 0.510 | 0.860 | 0.920 | 0.710 | 0.330 | 0.950 | 0.410 | 0.700 | 0.130 | 0.510 | 0.370 | 1.000 | 0.120 |
| **Q15** | 0.590 | 0.890 | 0.940 | 0.770 | 0.230 | 0.960 | 0.500 | 0.760 | 0.010 | 0.590 | 0.460 | 0.120 | 1.000 |

Determinant = 0.020

**Table 3. Total variance explained**

| **Component** | **Initial eigenvalues** | | |  | **Extraction sums of charges squared** | | | **Extraction sums of charges squared** | | |
| --- | --- | --- | --- | --- | --- | --- | --- | --- | --- | --- |
|  | **Total** | **(%) Variance** | **(%) Accumulated** |  | **Total** | **(%) Variance** | **(%) Accumulated** | **Total** | **(%) Variance** | **(%) Accumulated** |
| 1 | 4.341 | 33.396 | 33.396 |  | 4.341 | 33.396 | 33.396 | 2.371 | 18.241 | 18.241 |
| 2 | 1.343 | 10.328 | 43.723 |  | 1.343 | 10.328 | 43.723 | 2.315 | 17.810 | 36.051 |
| 3 | 1.140 | 8.772 | 52.495 |  | 1.140 | 8.772 | 52.495 | 1.914 | 14.720 | 50.771 |
| 4 | 1.091 | 8.391 | 60.886 |  | 1.091 | 8.391 | 60.886 | 1.315 | 10.115 | 60.886 |
| 5 | 0.870 | 6.689 | 67.575 |  |  |  |  |  |  |  |
| 6 | 0.830 | 6.384 | 73.959 |  |  |  |  |  |  |  |
| 7 | 0.713 | 5.488 | 79.447 |  |  |  |  |  |  |  |
| 8 | 0.648 | 4.985 | 84.432 |  |  |  |  |  |  |  |
| 9 | 0.568 | 4.370 | 88.802 |  |  |  |  |  |  |  |
| 10 | 0.465 | 3.580 | 92.381 |  |  |  |  |  |  |  |
| 11 | 0.418 | 3.213 | 95.595 |  |  |  |  |  |  |  |
| 12 | 0.361 | 2.774 | 98.369 |  |  |  |  |  |  |  |
| 13 | 0.212 | 1.631 | 100.000 |  |  |  |  |  |  |  |

*Extraction method: principal component analysis.*
